# Routine implementation costs of larviciding with *Bacillus thuringiensis israelensis* against malaria vectors in a district in rural Burkina Faso

**DOI:** 10.1186/s12936-016-1438-8

**Published:** 2016-07-22

**Authors:** Peter Dambach, Michael Schleicher, Hans-Christian Stahl, Issouf Traoré, Norbert Becker, Achim Kaiser, Ali Sié, Rainer Sauerborn

**Affiliations:** Institute of Public Health, University of Heidelberg, Heidelberg, Germany; South Asia Institute, University of Heidelberg, Heidelberg, Germany; Centre de Recherche en Santé de Nouna, Nouna, Burkina Faso; German Mosquito Control Association (KABS), Speyer, Germany; Centre for Organismal Studies, University of Heidelberg, Heidelberg, Germany

**Keywords:** Malaria, *Bacillus thuringiensis israelensis*, Vector control, Cost analysis, Larval source management, Burkina Faso, West Africa

## Abstract

**Background:**

The key tools in malaria control are early diagnosis and treatment of cases as well as vector control. Current strategies for malaria vector control in sub-Saharan Africa are largely based on long-lasting insecticide-treated nets (LLINs) and to a much smaller extent on indoor residual spraying (IRS). An additional tool in the fight against malaria vectors, larval source management (LSM), has not been used in sub-Saharan Africa on a wider scale since the abandonment of environmental spraying of DDT. Increasing concerns about limitations of LLINs and IRS and encouraging results from large larvicide-based LSM trials make a strong case for using biological larviciding as a complementary tool to existing control measures. Arguments that are often quoted against such a combined approach are the alleged high implementation costs of LSM. This study makes the first step to test this argument. The implementation costs of larval source management based on *Bacillus thuringiensis israelensis* (*Bti)* (strain AM65-52) spraying under different implementation scenarios were analysed in a rural health district in Burkina Faso.

**Methods:**

The analysis draws on detailed cost data gathered during a large-scale LSM intervention between 2013 and 2015. All 127 villages in the study setup were assigned to two treatment arms and one control group. Treatment either implied exhaustive spraying of all available water collections or targeted spraying of the 50 % most productive larval sources via remote-sensing derived and entomologically validated risk maps. Based on the cost reports from both intervention arms, the per capita programme costs were calculated under the assumption of covering the whole district with either intervention scenario. Cost calculations have been generalized by providing an adaptable cost formula. In addition, this study assesses the sensitivity of per capita programme costs with respect to changes in the underlying cost components.

**Results:**

The average annual per capita costs of exhaustive larviciding with *Bti* during the main malaria transmission period (June–October) in the Nouna health district were calculated to be US$ 1.05. When targeted spraying of the 50 % most productive larval sources is used instead, average annual per capita costs decrease by 27 % to US$ 0.77. Additionally, a high sensitivity of per capita programme costs against changes in total surface of potential larval sources and the number of spraying repetitions was found.

**Discussion:**

The per capita costs for larval source management interventions with *Bti* are roughly a third of the annual per capita expenditures for anti-malarial drugs and those for LLINs in Burkina Faso which are US$ 3.80 and 3.00, respectively. The average LSM costs compare to those of IRS and LLINs for sub-Saharan Africa. The authors argue that in such a setting LSM based on *Bti* spraying is within the range of affordable anti-malarial strategies and, consequently, should deserve more attention in practice. Future research includes a cost-benefit calculation, based on entomological and epidemiological data collected during the research project.

**Electronic supplementary material:**

The online version of this article (doi:10.1186/s12936-016-1438-8) contains supplementary material, which is available to authorized users.

## Background

Malaria is still the most common vector-borne disease in sub-Saharan Africa, although the global number of cases and deaths diminished considerably since the 2000s, by 18 and 48 %, respectively [1]. The former goal of global malaria eradication has been followed by an intensive public health focus on malaria control. Encouraged by recent control success, several countries now declared malaria elimination their policy target [2, 3]. Despite the historical success of malaria vector control through larval source management (LSM) [4], this method plays only a minor role on today’s sub-Saharan African policy agendas. Malaria vector control is mainly focused on strategies targeting adult mosquitoes. Long-lasting insecticide-treated nets (LLINs) are considered the first-line vector control intervention supported by indoor residual spraying (IRS) for selected settings. These methods, indeed, prove to be highly effective but challenges remain due to mosquito resistance to insecticides [5–8] and the presence of mosquitoes which feed and rest outdoors [9]. In this light, malaria vector control through larval source management shows high potential to work as a complementary strategy for effective malaria control systems.

Larvicides based on the mosquito-specific toxins produced by *Bacillus sphaericus (Bs)* and *Bacillus thuringiensis israelensis* (*Bti*) are promising tools for larval control. The selective nature of bacterial larvicides predestines them for the use in community settings. While essentially non-toxic in their natural state, once ingested and digested by immature mosquitoes, these toxins selectively kill the larvae of vulnerable mosquito species [10–12]. The advantage of targeting the larval stages of mosquito vectors is that they are immobile, and in most environmental settings present in well-defined and easily accessible places. Unlike adult mosquitoes they cannot change their behaviour to avoid control activities [13]. These larvicides operate on unique modes of action, allowing their use in resistance management programmes in vector control. In contrast to many other insecticides, to date no resistances to *Bti* have been observed in major malaria vectors [14].

*Bti* has been successfully used for decades in high-income countries, primarily against nuisance mosquitoes, such as floodwater mosquitoes [13]. However, in countries where vector borne diseases are most prevalent, LSM is still a widely neglected approach, and its application, with some exceptions, is limited to research settings. Nevertheless, this limited strand of empirical evidence shows LSM to be effective in reducing malaria transmission in various settings [15]. Most formulations of *Bti* show a strong lethal effect on larval populations that lasts between 1 and 2 weeks, leading to substantial reductions in adult vector densities [16–21]. Recent studies in East Africa have shown that larval control can reduce the abundance of malaria mosquito larvae and adult females by more than 90 % [22–24] and that combined with long-lasting impregnated nets (LLINs) a twofold reduction in new malaria infections can be achieved compared to LLINs alone [22]. Further evidence was generated in Eritrea, where LSM as an additional component in the vector control programme led to a reduction of malaria of more than 50 % [25].

Until now, one of the main factors preventing broader implementation of LSM programmes is the perception that they come with comparably high costs and require complex supply infrastructure. This is partly reflected by the World Health Organization’s stance towards LSM. While the WHO recently adapted its guidelines for *Bti*-based LSM and now recommends it for urban settings, rural areas are still considered ineligible due to alleged high programme costs [26]. To the authors´ knowledge, this cost argument has not been convincingly confirmed by empirical evidence, so far. Based on detailed cost information gathered during the implementation of a large-scale *Bti* based LSM programme (henceforth called LSM) in rural Burkina Faso this study assesses the per capita programme costs for two different treatment choices. Cost calculations have been made transparent and reproducible for other settings by providing an easily applicable and adaptable cost formula. In addition, this study takes a disaggregated look at the programme cost structure by assessing the sensitivity of per capita programme costs with respect to changes in the underlying cost components.

## Methods

### Study area

Home base of the project was Nouna, the capital town of the Kossi region, situated in the North Western part of Burkina Faso, close to the Mali border. Nouna itself with about 25.000 inhabitants is characterized as semi-urban while the surrounding villages are rural. The health district comprises 127 rural villages ranging in size from several hundred to a maximum of a few thousands inhabitants. The population covered by the health district aggregates to a total of 156,000 (2013) and is served by a total of 43 rural health centres, delivering basic medical treatment within their catchment area of on average 15 km radius [27]. Furthermore, those health centres serve as bases for health interventions, such as the distribution of insecticide-treated bed nets (ITNs) and vaccination campaigns.

Malaria transmission occurs throughout the year in Nouna, with a seasonal peak during the late rainy season between August and September. The entomological inoculation rate (EIR) which is defined as the number of infective bites per person per year amounted to 140 in 2002 for the area around Nouna [28] compared to an average of 700 for the Sahel region of West Africa [28, 29]. The study area has been subject to a wide set of malaria control interventions including the distribution of ITNs [30], intermittent preventive treatment in pregnancy (IPTp), and early diagnosis and treatment of malaria cases. Indoor residual spraying (IRS) is not implemented.

### Base study

The cost calculations are based on a prospective intervention trial in the Nouna Health District, implemented between 2013 and 2015 [31]. The larviciding intervention with *Bti* strain AM65-52 was performed during the rainy season from June to end October. While the district capital Nouna itself received full treatment, all 127 villages in the district were allocated into one of the following three equally sized study arms:

i. Treatment of all larval sources with *Bti* every 10 days (in the following called S1 scenario).
ii. Targeted treatment of the 50 % most productive larval sources, leaving the remaining 50 % least productive larval sources untreated (in the following called S2 scenario). The criterion “most productive” was based on entomologically validated risk maps, described elsewhere [32, 33].
iii. Untreated control group with no larviciding performed.

### Cost calculations

**Fig. 1:**
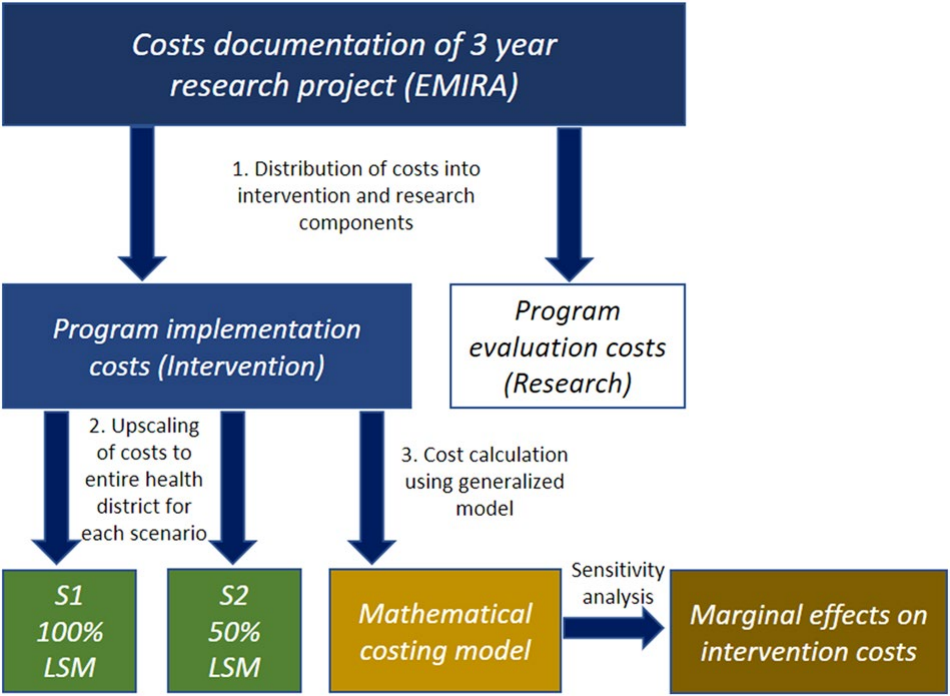
Process of cost finding within the research project, transformation to a routine implementation setting and upscaling of different larviciding strategies to the entire health district

The cost analysis consists of three steps, illustrated in Fig. 1. In step one, a comprehensive report on implementation costs based on detailed cost information gathered during the research project was established. In order to obtain full-scale per capita implementation costs, a second step, seized the costs for each of the two treatment choices (S1 and S2) under the assumption of having covered the whole health district and excluding research related costs. The third step, comprises a generalized cost model, providing a tool to transparently reproduce cost calculations for other settings. Based on this model, the programme cost sensitivity with respect to changes in underlying cost components was assessed.

### Assessment of project costs

Data were taken from the recorded project expenditures of the EMIRA project (Ecologic Malaria Reduction for Africa) [31]. Recorded cost information was translated into a spreadsheet-based cost-report, presented in the Appendix (Additional file 1: Table S1). For each component of the spraying programme, data were gathered from various sources. Larvicide consumption was recorded for each spray round at village level. Wages for fieldworkers were calculated based on the number of days worked for the programme, multiplied by a fixed daily rate. Costs for machinery, equipment and the larvicide, as well as their shipping costs were obtained from invoices and sales receipts from all project partners. A discount rate to calculate the annualized economic costs was not applied for two reasons. First, most of the capital costs (knapsack sprayers and field material) are only used during the project duration and their recovery value is difficult to determine. Second, some of the equipment used for the application of GPS, computer and satellite imagery was provided within the infrastructure of the host organization. Savings accrued from these in-kind benefits broadly outweigh potential cost reductions that may arise from writing down other assets to their annualized economic costs. The acquisition of satellite imagery and personnel costs for risk map creation were captured similarly. Costs were measured in the currency of the respective country (e.g. USA [US$], Germany/France [€], Burkina Faso [FCFA], and converted into US$ using a 2013 through 2015 average exchange rate of 1US$ = 0.82 € = 531 FCFA (oanda.com). Consumables and utilization of existing resources at the research centre (cars and drivers, etc.) were recorded using the research centre´s charges and allowances. Opportunity costs were excluded, (missed potential gains from alternative interventions when one intervention has been chosen) and the focus put on financial costs only.

In a further step, all research-related costs such as impact evaluation and university salaries that would not incur in a routine intervention were excluded. This step allowed for a cost comparison with other vector control approaches such as LLINs and IRS. Costs of international staff were replaced with those for local personnel where possible. External specialists for LSM, remote sensing and risk map creation were kept since those might not be locally available in comparable settings.

### Scenario upscaling

Accrued project implementation costs were recorded for both treatment scenarios, which accounted for roughly one-third of the study population each. These costs were extrapolated to the entire health district comprising 127 rural villages and the district capital. Based on practical experience gathered during implementation, the following adjustments were performed before conducting the scale-up calculations. Due to high heterogeneity in size and number of larval breeding sites across villages, an hour-based pay scheme was introduced to account for the varying workloads for spray personnel. Equally, the fixed number of two sprayers per village employed during the research project was adjusted to one person who is paid for the hours worked. For a small percentage of spray-intensive villages additional spray personnel was budgeted. This means the personnel needs between different treatment scenarios are expressed through workload and not by the number of people *per se*.

An estimated total of 150 persons would be needed to cover the entire health district including the district capital. This roughly translates into one person per village plus 15 additional sprayers reserved for some villages which show more than 1.5 hectares of larval sources, which is roughly the surface that can be treated by one person per day. These adjustments differ from the setup in the research project, where the required workforce was overestimated for most villages and two fieldworkers with spraying equipment were employed per village. According to the personnel adjustments from above a total of 25 supervisors would be needed to ensure quality control of the larviciding actions by revisiting larval sources and sampling larvae.

### Modelling of programme costs

To allow for a more generalized calculation of implementation costs for different settings, we derived a cost formula that explains the relationship between programme costs and its components. The contribution from such an exercise is twofold: First, it sheds light into the cost structure of a large-scale LSM programme in the highly relevant context of a malaria stricken sub-Saharan African country. Second, the formula provides a fair estimate of per capita implementation costs for *Bti*-based LSM, using reference values from the EMIRA project, from previous findings and from literature as input variables (Table 1). Thus, the formula easily accounts for a wide range of implementation scenarios, such as a changed larvicide market price or labour wage rate. By controlling for different geographic conditions (e.g. the duration of the rainy season), different *Bti* formulations (e.g. re-treatment interval, larvicide concentration) and treatment productivity (e.g. application mode, distribution of breeding sites) the presented cost-model can be regarded as a suitable ex-ante assessment tool for upcoming LSM interventions.

**Table 1:** Baseline, minimum and maximum values for parameters that influence project costs

Variable name	Unit	Description	Baseline	Min	Max	Determination of range
C_BTI_	US$ per kg	Price of larvicide including freight costs	51.00	43.00	54.00	R1
BtiConc	Kg per hectar	Larvicide concentration	0.35	0.30	0.40	R2
Repetitions	Times per year	Number of spray rounds per year	15	12	18	R3
TotalSurface	Hectar	Total surface to treat within the region of intervention	240	192	288	±20 %
CoveredSurface	Discrete multiplier	Percentage of possible larval sources that receives treatment (exhaustive treatment = 100 %, targeted control = 50 %)	1 in S1 scenario 0.5 in S2 scenario			Depending on defined scenario.
C_MAT_	US$ per person	Costs for knapsack sprayers and protective clothes per sprayer including freight costs	77	62	92	±20 %
SprayerProductivity	Hectar per hour	Surface that one sprayer can treat per hour	0.25	0.20	0.30	±20 %
HoursDay	Hours per working day	Number of hours that spray teams work per day	6	5	7	Common workloads occurring during the research project
SprayerWage	US$ per hour	Hourly wage	0.25	0.20	0.30	±20 %
SupervisorProductivity	Hectar per hour	Surface that one supervisor can control per hour (larvae dipping)	0.5	0.4	0.6	±20 %
SupervisorWage	US$ per hour	Hourly wage	3.5	2.8	4.2	±20 %
SampleSurface	Discrete multiplier	Percentage of treated surface that the supervisors check with larvae dipping for quality control	0.2	0.1	0.3	Limits considered to be still sound (lower limit) and economically justifiable (upper limit)
C_PM_	US$	Cost for project manager	9800	7840	11,760	±20 %
C_LSM_	US$	Cost for LSM specialist	2800	2240	3360	±20 %
C_MSZ_	US$	Cost for community sensitization	1067	854	1280	±20 %
C_TRAIN_	US$	Cost for training events	2600	2080	3120	±20 %
C_EQP_	US$	Cost for mapping equipment	833	666	1000	±20 %
C_SOURCE_	US$	Mapping of larval sources	600	480	720	±20 %
C_GIS_	US$	GIS map creation	1400	1120	1680	±20 %
C_SAT_	US$	Satellite imagery	550	440	660	±20 %
C_RISK_	US$	Risk map creation	933	746	1120	±20 %
C_TRANSROUND_	US$ per repetition	Transport costs, gas and allowance for fieldworkers	300	240	360	±20 %
M_OVERHEAD_	Discrete multiplier	Percentual share added to the subtotal costs	0.1	0.1	0.15	Common additions for overheads in research projects

The baseline values represent the average settings that underlay the cost calculation

As shown in Eq. (1) the total programme costs were disaggregated (C_TOTAL_) into seven cost components. Each component in turn, is expressed as a function of its underlying cost variables and will be further explained in the following.

1 $${\text{C}}_{\text{TOTAL}} = {\text{C}}_{\text{LARV}} + {\text{C}}_{\text{SPRAY}} + {\text{C}}_{\text{QUAL}} + {\text{C}}_{\text{PMT}} + {\text{C}}_{\text{MAP}} + {\text{C}}_{\text{TRANS}} + {\text{C}}_{\text{OVER}}$$

### Larvicide needs and spraying apparel (Eq. 2)

Total larvicide-related costs (C_LARV_) contain the cost for obtaining the needed quantity of larvicide (*C*_*BTI*_) as well as corresponding spraying material (*C*_*BTIMAT*_) and are presented in Eq. (2).

First, C_LARV_ depends on the exogenously given larvicide market price (*C*_*PBTI*_) measured in US$ per kg. Expenses for shipping the product from the US to Burkina Faso, including insurance and clearance fees (Manufacturer Valent Biosciences, exchange rates US$ to € averaged 2013–2015) were included. Storage of the product once it arrived at district level was considered under operations costs and overheads. The spraying interventions were run with VectoBac^®^ WG, a water dispersible granule formulation of *Bacillus thuringiensis israelensis* strain AM65-52 from Valent BioSciences Corp., Illinois, USA with a potency of 3000 ITU/mg (International Toxic Units).

Second, larvicide-related costs depend on the ultimate choice of the utilized *Bti* concentration (*BtiConc*). In our setting, the *Bti* granules were suspended in water prior to usage and dispersed in stagnant environmental water bodies using knapsack sprayers (Model: 3595 INOX, Mesto Corp., Freiberg, Germany).

Third, the amount of larvicide needed depends highly on the duration of the intervention within a year, which for its part is driven by the onset and cessation of the rainy season. Moreover, implications for larvicide consumption arise from the treatment interval, the number of days one waits until the next spraying is conducted. When deciding on the optimal interval length the following trade-off arises: from a cost perspective the interval should be maximally stretched while shorter treatment intervals ensure that a maximum of larvae are hindered to develop into adult vector mosquitoes [17]. Both factors, duration and treatment interval are reflected by the variable *Repetitions*, the number of treatments per year for every eligible unit.

Another factor exerting influence is the buffer radius chosen to be treated around a village, accounting for the mosquitoes’ flight range and their possible infiltration from outside into treated areas. The buffer radius is not separately included within the cost formula but is represented through the total surface of breeding sites in the vicinity of villages (*TotalSurface*). The variable *CoveredSurface* accounts for both treatment scenarios, the 100 % (*CoveredSurface* = 1) and the 50 % (*CoveredSurface* = 0.5) treatment.

Finally, the costs for corresponding spraying material are added to the equation where the number of sprayers is approximated and multiplied with the costs of knapsack sprayer equipment per person (*C*_*BTIMAT*_). The latter were accounted similarly to *C*_*BTI*_, but with transport from Germany.

2 $$\begin{aligned} {\text{C}}_{\text{LARV}} &= {\text{C}}_{\text{BTI}} + {\text{C}}_{\text{BTIMAT}}\\ & = \left( {{\text{C}}_{\text{PBTI}} *{\text{ BtiConc }}*{\text{ Repetitions }}*{\text{ TotalSurface }}*{\text{ CoveredSurface}}} \right)\\ & \quad + \left( {{\text{C}}_{\text{PMAT}} *\frac{TotalSurface}{Sprayer\Pr oductivity*Hours\;Per\;Day}} \right) \\ \end{aligned}$$

### Staff requirements and costs (Eqs. 3–5)

A large-scale LSM programme requires the employment of different types of personnel. For project management, risk map creation, and entomological training highly trained professionals are required, while for spraying activities laypersons from the villages can be hired and trained within a short time. Calculation of personnel costs is based on the research centre´s guidelines, which follow recommendations by the ministry of health and may be similar within the region. Based on the experience, with a 5-month implementation period, spray personnel would have to work approximately only on 15 days in total.

As it can be seen in Eq. (3) costs for spraying activities (*C*_*SPRAY*_) can be described as product of four factors. The first four factors determine the total amount of working hours needed for spraying which is then multiplied with the hourly rate.

3 $${\text{C}}_{\text{SPRAY}} = {\text{Repetitions}}*\frac{TotalSurface}{Sprayer\Pr oductivity} *{\text{ Surface }}*{\text{ CoveredSurface }}*{\text{ SprayerWage}}$$

The costs for quality control (*C*_*QUAL*_) are expressed in Eq. (4). In this setting, the corresponding effort for sending supervisors to the field amounts to 30–35 days per season. In contrast to spraying personnel supervisors are differently qualified and follow another project plan with spot tests at several treatment units or villages per day. Thus, Eq. (4) shows a similar structure to Eq. (3) but assumes different values for labor productivity (*SupervisorProductivity*) and hourly rates (*SupervisorWage*). The percentage of total surface selected for quality control spot tests (*SampleSurface*) is variable and can be chosen by the programme manager. Here, a tradeoff is faced between high quality control standards and lower personnel costs.

4 $$\begin{aligned} {\text{C}}_{\text{QUAL}} &= {\text{Repetitions}}*\frac{TotalSurface}{Supervisor\Pr oductivity} \\ & \quad *{\text{ SupervisorWage}} *{\text{ Surface }}*{\text{ CoveredSurface }}*{\text{ SampleSurface}} \\ \end{aligned}$$

Costs for project management and training (*C*_*PMT*_) are presented in Eq. (5). The personnel costs for the project manager (*C*_*PM*_), as well as those of the entomological technicians (*C*_*LSM*_) were calculated as monthly salaries multiplied by the number of months involved in the project. The costs for community sensitization (*C*_*MSZ*_) and training events (*C*_*TRAIN*_) are rather dependent on their complexity and duration than on the sheer number of people and are therefore additively linked.

5 $${\text{C}}_{\text{PMT}} = {\text{C}}_{\text{PM}} + {\text{C}}_{\text{LSM}} + {\text{C}}_{\text{MSZ}} + {\text{C}}_{\text{TRAIN}}$$

### Mapping and risk map creation (Eq. 6)

Two different types of maps were used in the underlying intervention study, depending on the chosen intervention scenario. Consequently, costs for mapping and risk map creation differed by treatment scenario and are presented in Eqs. (6) and (7). Both treatment choices required mapping of larval sources prior to map creation. While for the S1 scenario all breeding sites had to be mapped individually to obtain a map with their positions (*C*_*SOURCE*_), the S2 scenario needed only mapping of a defined percentage of breeding sites including their vector larvae productivity (*C*_*RISK*_). Regarding the latter, satellite image analysis was performed to generate a risk map of the villages in question (C_GIS_). The costs for the S2 scenario included purchasing of SPOT 5 satellite imagery from ASTRIUM Geo Information Services (*C*_*SAT*_), while GPS handheld devices and software licenses were required for both scenarios (*C*_*EQP*_).

6 $${\text{S1 scenario }}\left( {100{\text{ percent treatment}}} \right){\text{: }}{{\text{C}}_{{\text{MAPS1}}}} = {{\text{C}}_{{\text{EQP}}}} + {{\text{C}}_{{\text{SOURCE}}}}$$

7 $${\text{S2 scenario }}\left( {50{\text{ percent treatment}}} \right){\text{: }}{{\text{C}}_{{\text{MAPS2}}}} = {{\text{C}}_{{\text{EQP}}}} + {{\text{C}}_{{\text{GIS}}}} + {{\text{C}}_{{\text{SAT}}}} + {{\text{C}}_{{\text{RISK}}}}$$

### Transport costs and overheads (Eqs. 7 and 8)

Transport costs (*C*_*TRANS*_), presented in Eq. (8), are given as the product of repetitions and cumulated transport costs per treatment round (*C*_*TRANSROUND*_). The latter includes expenditures for the use of cars and motorbikes. It equally covers proportionate salaries of drivers and allowances. Transport costs mainly occur during field missions to the villages where spraying is performed to deliver the larvicide, hold sensitization meetings, and locally coordinate with spray teams.

8 $${\text{C}}_{\text{TRANS}} = {\text{Repetitions }}*{\text{ C}}_{\text{TRANSROUND}}$$

Overheads (*C*_*OVERSUBTOTAL*_) comprise office and storage space requirements, insurance, security, and other office support requirements, such as stationary, printing, communication and field mission coordination. Equally consumables not represented as separate budget items and were included within overheads. Remaining personnel expenses spent on short-term consultancies were included in overheads (e.g. secretary, accountant).

9 $${\text{C}}_{\text{OVERSUBTOTAL}} = {\text{Subtotal Costs }}*{\text{ M}}_{\text{OVERHEAD}}$$

### Sensitivity analysis of per capita intervention costs

The last part of our cost calculation approach describes the sensitivity analysis that builds on the *Bti* cost model (0). The variables chosen were either fixed a priori within the programme protocol, e.g. the surface of the treatment buffer around villages, the utilized *Bti* concentration, and the treatment interval (in the following called endogenous factors) or they were subject to local environmental settings, e.g. the average total surface of larval sources per village (in the following called exogenous factors).

Based on the reference values from our intervention, from literature and from own preceding field studies [17, 31], a reasonable interval with lower and upper limits for each variable was determined. Since the underlying baseline values were taken from effectively accrued costs, no confidence intervals were available to fix the minimum and maximum values. Instead we used a 20 % fluctuation range for most personnel and material costs. The variable population density was excluded from the formula. This is because those data are commonly only available for a predefined region but hardly for an area of treatment in and around villages. Instead the formula facilitates the calculation of total programme costs, which can afterwards easily be divided by the population domiciled in the villages that are covered by spraying activities.

*R1* The price for *Bti* refers to the formulation and manufacturer used within the underlying field study (Vectobac^®^ WG, formerly WDG, Valent Biosciences Corp.). With the same product, semi-terrain studies on lethal dosages were performed [17]. The costs for the larvicide were combined with the costs for different freight options. The baseline and maximum values represent typical airfreight costs from the US to West African countries. Total airfreight costs increase virtually linearly with the amount of larvicide ordered. The minimum value represents the cheapest offer by sea cargo which is usually not bound to weight but to volume, e.g. per container space. The volatility of the dollar to euro exchange rate over the last 3 years was included into our minimum and maximum value calculations since many West African currencies are pegged to the Euro. Average exchange rates for 2013, 2014 and 2015 were used.

*R2* The efficacy of *Bti* formulations against *Culicidae* larvae depends on various factors, including water pollution, water depth, water temperature and others. The baseline value corresponds to the concentration used during the study, the minimum value showed efficacy in semi-field trials in the region and could still be effective in achieving >95 % lethality on mosquito larvae. The upper threshold might be needed if Culex mosquitoes should become a target, which often breed in heavily polluted breeding sites.

*R3* The number of repetitions is the product of the period of intervention (months per year) and the spraying frequency (spray rounds per month). It depends on the duration of the rainy season and the persistence of larval breeding grounds. The given minimum and maximum values do not only attribute for the interannual variations in the onset and duration of the rainy season [34] but reflect a possible shift of the region of implementation further North or South by some 100 km. The number of repetitions can be equally influenced by the use of other *Bti* formulations or differences in temperature which will determine the spraying frequency via their influence on larval development.

## Results

### Accrued costs for LSM in the underlying research setting

All expenditures that accrued during the 3-year project were recorded and assigned to respective cost categories. The overall costs of the project as it was performed in the field amounted to US$ 563,690, including research related costs (see Additional file 1: Table S1). Those research related costs are provided by Additional file 1: Table S1 to increase transparency and to facilitate replicability. Research related costs were excluded during the following upscaling process. During the two intervention years of the study a total of 1800 kilograms of *Bti* powder were deployed, inducing expenditures of roughly US$ 73,000, including freight costs, this figure increases to US$ 94,000, accounting for 16 % of the total costs of the research project. Within the research setting personnel costs amounted to roughly US$ 322,000, which represents almost two-third of total programme costs. More than half of those costs were consumed by the salary of an external scientific coordinator. Additional US$ 50,000 were used for the implementation of impact evaluation tools. Research-related costs amounted roughly to US$ 257,000 which represents 45 % of total programme costs.; they would not incur during a routine implementation of larviciding activities.

### Upscaling of per capita intervention costs

The programme costs were calculated under the assumption of covering the whole health district and running the programme for 3 years for both treatments (S1 and S2) (see Table 2). Three different costing results are provided: Column (1) shows, the costs per person protected for the first intervention year. Column (2) presents costs for running the project an additional year while column (3) gives the average costs over the assumed programme duration of 3 years. The latter allows the best estimation of incurring cost per year and the comparison with other vector control methods, and are hence used for further consideration.

**Table 2:** Costs for LSM in US$ in the Nouna health district comprising 127 rural villages and the semi-urban district capital

	Implementation year		Following program year		Average annual costs over 3 years		Proportion of total costs (%)	
	S1	S2	S1	S2	S1	S2	S1	S2
Recurrent costs (personnel)								
Project manager Burkina Faso	9.800	9.800	9.800	9.800	9.800	9.800		
LSM specialist	4.200	4.200	2.100	2.100	2.800	2.800		
Entomologic technicians	4.800	4.800	4.800	4.800	4.800	4.800		
GIS specialist		4.200				1.400		
Larviciding personnel	28.000	22.500	28.000	22.500	28.000	22.500		
Larviciding supervisors	7.750	7.750	7.750	7.750	7.750	7.750		
Subtotal	54.550	53.250	52.450	46.950	53.150	49.050	35.9	44.9
Consumables								
Larvicide	56.000	28.000	56.000	28.000	56.000	28.000		
Air freight larvicide	14.500	7.250	14.500	7.250	14.500	7.250		
Subtotal	70.500	35.250	70.500	35.250	70.500	35.250	47.6	32.2
Transport costs								
In country travel and field work	5.700	6.900	5.700	5.700	5.700	5.700		
Subtotal	5.700	6.900	5.700	5.700	5.700	5.700	3.8	5.2
Activities								
Staff training	3.800	3.800	2.000	2.000	2.600	2.600		
Community sensitization	3.200	3.200			1.067	1.067		
Mapping of larval sources	1.800				600			
Mapping for risk map creation		2.800				933		
Subtotal	8.800	9.800	2.000	2.000	4.267	4.600	2.9	4.2
Capital costs								
Knapsack sprayers	36.000	36.000			12.000	12.000		
Air freight knapsack sprayers	7.700	7.700						
GPS, computer, equipment	2.500	2.500			833	833		
Satellite imagery		1.650				550		
Protective clothes	5.300	3.975			1.767	1.325		
Subtotal	51.500	51.825			14.600	14.708	9.9	13.5
Total	191.050	157.025	130.650	89.900	148.217	109.308		
Overheads 10 %	19.105	15.703	13.065	8.990	14.822	10.931		
Total program costs	210.155	172.728	143.715	98.890	163.038	120.239		
Costs per person protected	1.35	1.11	0.92	0.63	1.05	0.77		

Costs per person protected are based on a 2013 midyear population of 156.000 inhabitants. Calculations based on 2013 costs for material and personnel using a 2013–2015 average exchange rate of US$ 1 = € 0.82

The total annual costs averaged over 3 years for the S1 scenario were estimated at US$ 163,038 to cover 156,000 people in a mostly rural area with villages distributed over 4200 km^2^. The total annual costs for the S2 scenario amounted to US$ 120,239. The costs per person protected per year were calculated to be US$ 1.05 and US$ 0.77 for the S1 and S2 scenarios respectively. Over 3 years, the recurrent costs comprised about 80 % of the total programme costs for both S1 and S2. The highest share of costs was attributable to larvicide within the S1 scenario with 41.6 % of total costs while for the S2 scenario larvicide and its transport only amounted to 28.0 % of total costs. Personnel costs made up 34.7 % of total costs in the S1 scenario while their proportion was markedly higher in the S2 scenario with 42.7 %.

### Sensitivity analysis of per capita intervention costs

The sensitivity analysis evaluates the responsiveness of the programme’s *per capita* costs towards changes in major cost component variables. Results are interpreted in a *ceteris paribus* fashion; A component’s effect on total programme costs is assessed while keeping all other cost components constant. The selection of input variables is directly based on the *Bti* cost model introduced in the above section (Eq. 1). Based on its magnitude, relevance and simplicity of interpretation the following five variables are included in the sensitivity analysis; *TotalSurface, Repetitions, BtiConcentration, SprayerWage, BtiCost*.

**Fig. 2:**
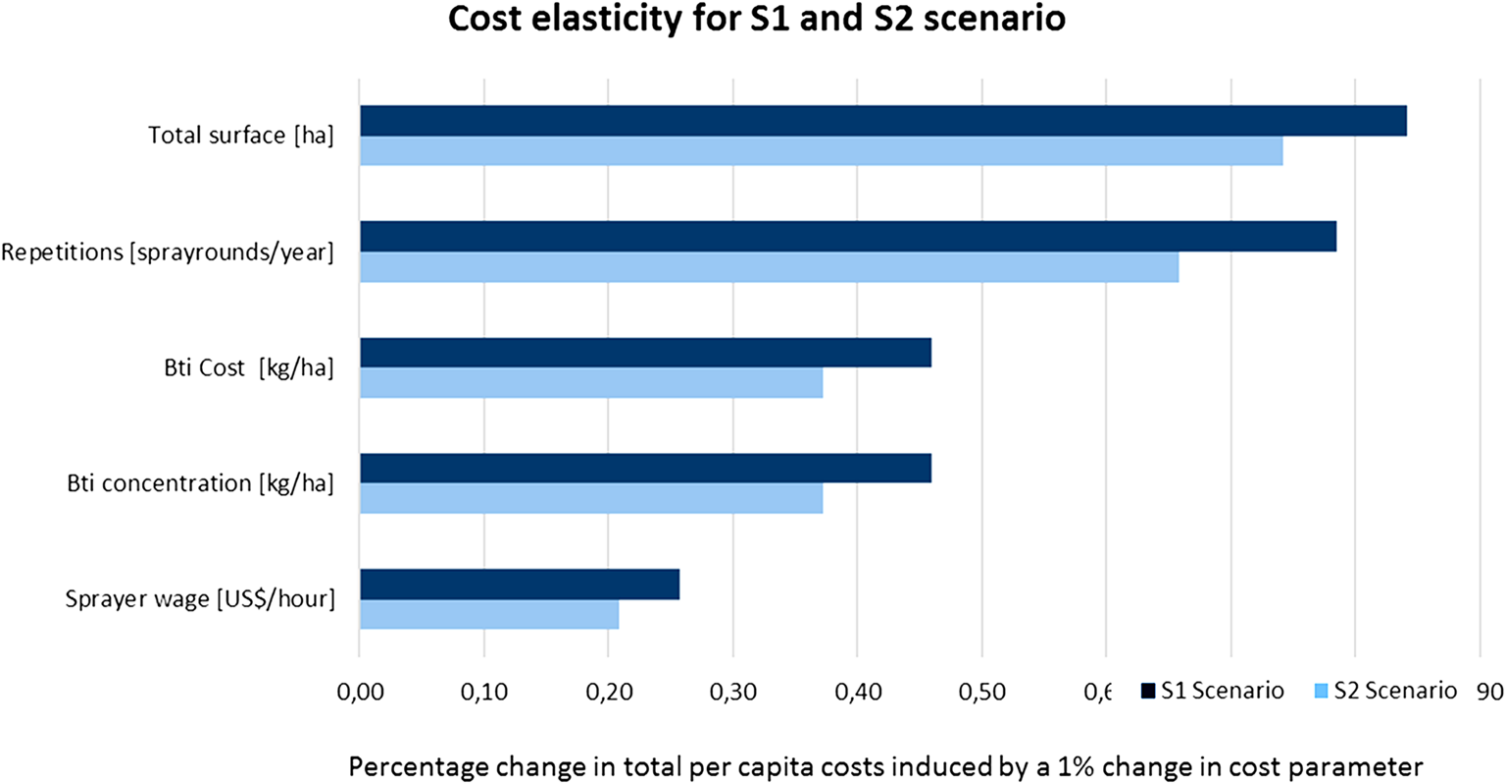
Elasticity of cost factors for the S1 and S2 scenario. The diagram shows the percentage change of costs per person and year protected if the cost factor varies by 1 %

The first part of the analysis looks into the elasticity of programme costs; the percentage change in programme costs when the cost of only one component increases by 1 %. Figure 2 illustrates the corresponding results for both scenarios. Since elasticities behave symmetrically it is sufficient to only depict one direction of the cost component change. Two exogenous factors, *total surface* and *sprayer wage* determine the boundaries of cost elasticity. For scenario 1, a 1 % increase in *total surface* leads to a 0.84 % cost increase while a 1 % increase in *sprayer* wage leads only to a 0.25 % cost increase. Increasing the *number of repetitions* by 1 % still leads to substantial increases in programme costs of about 0.78 and 0.65 %, for the first and second scenario respectively. Programme costs show moderate changes related to an increase in the price and utilized concentration of *Bti* of about 0.45 and 0.38 % in S1 and S2, respectively.

**Fig. 3:**
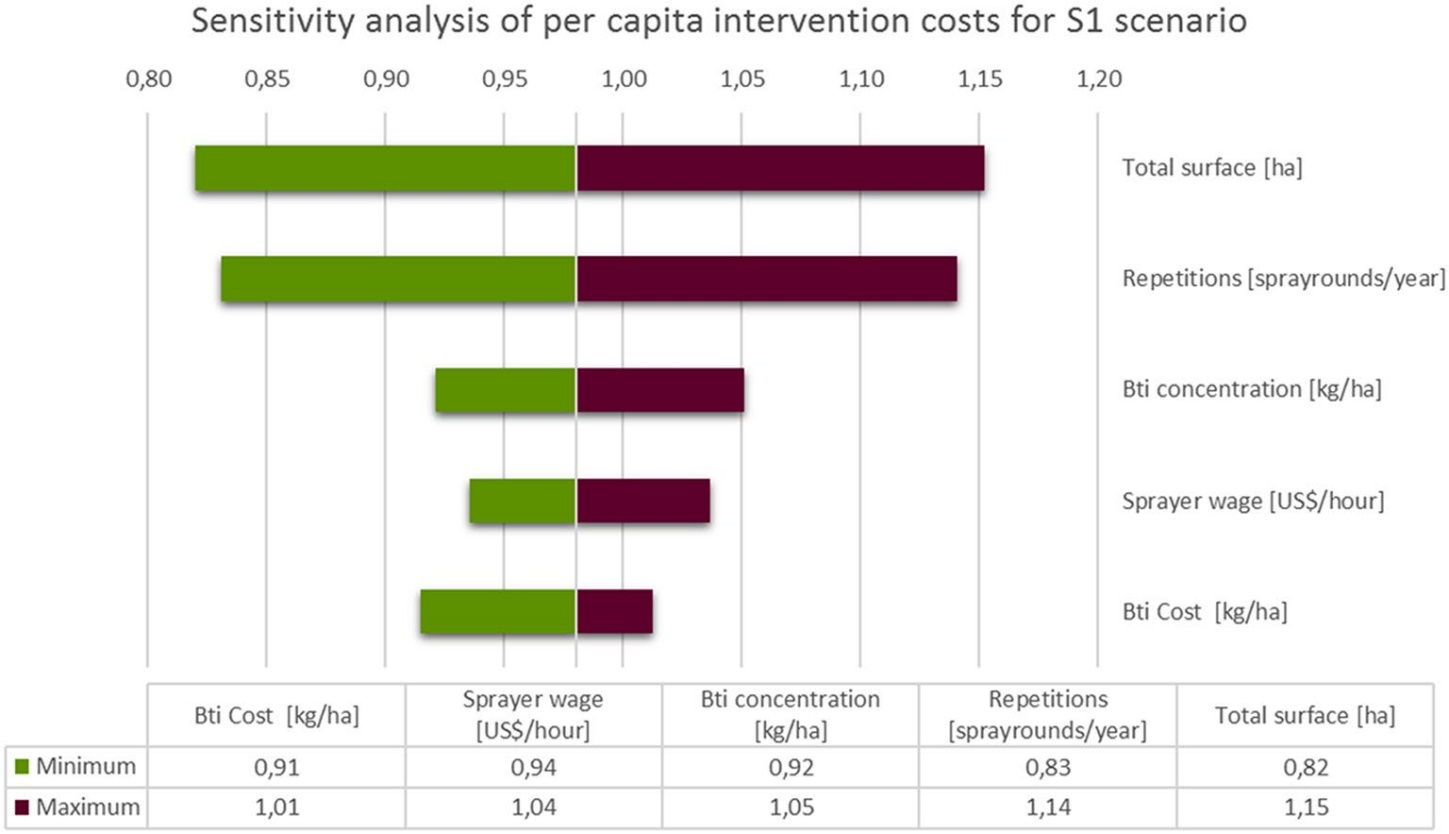
Sensitivity analysis of costs per person and year protected for S1. Dependencies of costs on deviation from the baseline value are shown for the five most influential parameters. Intervals for the depicted cost components are: CBTI: −16/6 %; SprayerWage: −20/20 %; BtiConc: −14/14 %; Repetitions: −20/20 %; TotalSurface: −20/20 %

**Fig. 4:**
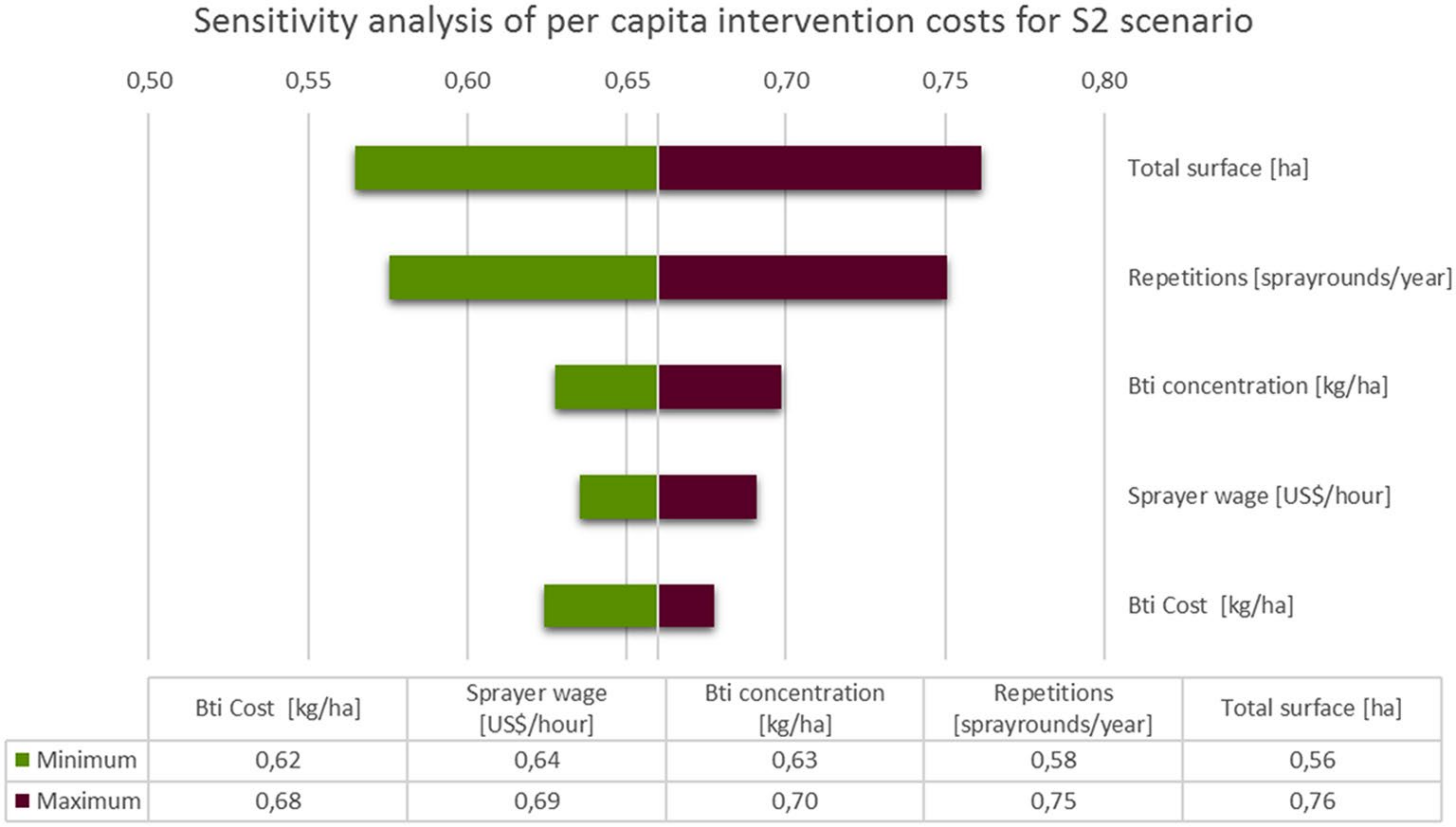
Sensitivity analysis of costs per person and year protected for S2. Dependencies of costs on deviation from the baseline value are shown for the five most influential parameters. Intervals for the depicted cost components are: CBTI: −16/6 %; SprayerWage: −20/20 %; BtiConc: −14/14 %; Repetitions: −20/20 %; TotalSurface: −20/20 %

Further, there is interest how absolute values of per capita programme costs change with respect to the determined interval-width of component costs. Such an illustration in absolute terms contains useful information about the directly accrued cost changes one would expect in the field. For both scenarios this is presented in Figs. 3 and 4, respectively. Relative to the elasticity representation it can be observed that *Bti* costs now show a relatively smaller effect since the current level of *Bti* prices is judged to be quite high, already. The latter also explains the relatively high swing to the left of the baseline value. In absolute terms, increasing *total surface* by 20 % (from 240 to 288 hectares) leads to an increase in total per capita programme costs of about US$ 0.17. High sensitivity equally originates from the number of spray rounds performed during a year. An extension or shortening from the standard 15 rounds of larvicide application by 20 % (equaling 1 month of intervention), will increase or lower the intervention costs by roughly US$ 015 (US$ 0.09 for S2). The following parameters have much less impact on the intervention costs when varying within their predefined borders. All parameters that have a lower impact on the per capita costs than 1 % are not shown. The modelled total programme costs were divided by the number of inhabitants (156,000) that would be covered in a programme run in the complete health district.

When substituting the hourly payment scheme of the costing model with a daily payment scheme based on the number of spray rounds performed, calculated costs increase by about 14 % (23 % for S2). A daily payment scheme is easier to implement in areas where one can find delimited villages rather than a coherent treatment area. In such a setting the amount of spraying apparel increases disproportionally since equipment will remain within one village even if there are only few breeding sites to treat. In a strictly village-based treatment scheme the difference between a S1 and a S2 scenario reduces because the minimum requirement of sprayers per village is one person. For more coherent settlements or areas with villages close to each other though, an hourly treatment scheme seems more appropriate to calculate costs, since spray personnel and knapsack sprayers can be distributed as needed within that region.

## Discussion

It was found that the implementation of a comprehensive LSM coverage of the Nouna health district, would incur annual per capita costs of US$ 1.05 (US$ 0.77 for S2 scenario) over a project lifetime of 3 years. Other studies [23, 35, 36] found similar values ranging between US$ 0.90 and 2.50, with the general tendency of lower per capita costs in urban settings. In the Upper-Rhine valley in Germany a mosquito control programme using *Bti*-based routine LSM has been carried out on a large-scale operational basis since 1992. It’s per capita costs of US$ 1.18, are in a comparable range [37] even though institutional, economic and environmental conditions are highly different. In this setting of a developed country, high population density and high degree of mechanization outweigh the relatively high costs for salaries. The calculated per capita intervention costs for Bti-based LSM in Burkina Faso compare with the lower limit of costs for indoor residual spraying, which range from US$ 0.88–4.94 per person protected [38]. They are also slightly below the costs for long-lasting impregnated bed nets (LLINs) in sub-Saharan Africa, which are in the range of US$ 1.38–1.90 [39]. The per capita costs for larviciding in the underlying setting were significantly lower than those for conventional ITNs which range between US$ 1.21 in Eritrea and US$ 6.05 in Senegal [38, 39] and were found to have median standardized costs of US$ 2.20 per year [40]. Compared to the national per capita expenditures in Burkina Faso for LLINs and anti-malarial drugs which amount to US$ 3.00 and US$ 3.80, respectively [41], costs for larviciding were significantly lower.

The programme’s per capita costs were calculated in two different ways. The method of upscaling is naturally bound strongly to expenditures that were seized during the research project. The *Bti* cost model tries to unfold the programme’s underlying cost structure and, at the same time, provide an ex-ante assessment tool, suitable for LSM interventions in similar settings. In contrary to the up-scaled costs, the cost model generalizes more, e.g. the needed workforce is broken down on hourly base. As a result, per capita intervention costs slightly differ between both approaches. For the above comparison with other control measures we used the values calculated during the upscaling process.

The costs per person protected are driven mainly by two factors, the water surface to be treated and the number of inhabitants that benefit from the intervention in the respective village. Furthermore, the costs of larviciding depend on the region's ecology, e.g. the number of months with persisting larval sources in the environment. In the setting of North Western Burkina Faso, the highest transmission of malaria takes place during the 5 months of the rainy season (June through October). In areas with two rainy seasons or year round rainfall, the costs of a larviciding programme are likely to increase, while in areas with a shortened rainy season costs will decrease. Climatic changes might equally show impact on the required duration of larviciding activities in future [42].

There are limitations to the economically efficient application of LSM for areas with a high annual malaria transmission and vast areas covered by surface water [20, 43–45]. However, in the light of recent control efforts with LLINs, more areas show reduced malaria transmission during an increased number of months [25, 36]. This would make the additional implementation of larvicide-based LSM economically suitable even in previously unsuitable regions, provided it is part of integrated malaria control, i.e. applied together with medical treatment, LLINs and IRS. Although the WHO recommends that “larviciding should be considered for malaria control (together with or without other interventions) only in areas where the larval habitats are few, fixed and findable” [26] the Cochrane Review of Tusting et al. [15] summarized evidence for high success at reasonable costs even for regions where the WHO criteria are not fulfilled, which is in line with the results of this study.

The targeted treatment strategy might prove to be especially useful in settings with a high number of larval sources and a prolonged duration of vector abundance and hence malaria transmission. Here, reducing the area to be sprayed by targeting only highly productive water bodies using remote sensing could be an effective and cost-reducing option. It is known that not all water bodies are appropriate for malaria vector development [44, 46] and costs can be reduced if only those with increased larval productivity are treated [32, 33]. In this research setup, a threshold of 50 % of the expected most productive water bodies to treat within the larviciding scenario S2 was chosen. Though this threshold might be increased or lowered in view of different environmental conditions, it should ideally follow the absolute larval productivity per surface.

We found the S2 scenario to have roughly 26 % lower costs of treatment than the S1 scenario, primarily due to savings on larvicide and personnel costs. Results from the EMIRA study show an average mosquito reduction in the S2 study arm of roughly 65 %, compared to 80 % in the S1 scenario (Dambach, unpublished data). At a later stage, savings in treatment costs have to be thoroughly evaluated against the lower achieved reductions in vector abundance. Risk map based larviciding is particularly interesting for settings with a large number of environmental larval sources, since in such environments costs for larvicide and workforce increase disproportionally under a full coverage scenario. On the other hand, this requires a high prediction accuracy of the risk model and the existence of larval sources with low or no larval productivity [47]. In settings where the majority of water bodies is infested with vector larvae, like it was the case in the study region, exhaustive larviciding would be the better choice. The deductions compare to results of some other studies that found high degrees of larval infestation regardless of the type of breeding site [48, 49]. In other areas with high heterogeneity in breeding site infestation, targeted interventions might be an opportunity to cut down on personnel costs as another large-scale trial has shown for dengue [50]. Technological developments such as new larvicide formulations with longer residual effects through encapsulation and via the combination with other larvicides will substantially reduce treatment costs. Those would drop by roughly 25 % if the treatment intervals were extended from 10 to 14 days. Innovations such as airborne dispersal via unmanned aerial vehicles might further reduce programme costs [51].

The calculations presented here are the basis for a cost-effectiveness analysis to come, evaluating the entomologic and health impact indicators [31]. A crucial factor for a cost-effectiveness analysis at a later stage is to measure the attributable effect of LSM on health outcome indicators. Cost calculations were based on the project duration of 3 years, although several items of the capital costs might have a significantly longer service life. If implemented as a routine vector control measure costs for personnel training and sensitization would equally decline.

Despite differences in climate, environment and population densities in other parts of Burkina Faso and neighboring countries, the presented findings can be a useful reference to calculate budget needs for similar implementations, be it regional or nationwide. Those would add onto the national health expenditures of US$ 39.00 per person and year [41]. In developing countries such as Burkina Faso though, to date such an intervention, even if cost effective in terms of its protection against malaria, needs funding from external donors. In the light of our findings one of the main points of criticism towards LSM, their perceived high costs, should be revised for a number of settings. The results show that LSM based on *Bti* spraying can be an affordable complementary approach for integrated malaria control. Further in-depth research is needed to more comprehensively compare the programme costs and malaria control benefits across different approaches. With respect to this intervention study, such an analysis is planned for future research and will assess the cost-effectiveness based on parasitaemia and malaria-related morbidity and mortality.

There are several strengths and limitations to this study. To the authors' knowledge this is the first study that empirically assessed *Bti* larviciding costs in West Africa based on a large-scale LSM project. The presented cost report draws on effectively accrued expenses and serves as a reliable indicator for projects in similar settings. On the other hand, this very detailed calculation bears limitations. If the programme costs are to be compared with those from other settings, e.g. east Africa or Asia, differences in organizational frameworks and their guidelines such as from the ministry of health and locally performing institutions are likely to substantially affect the intervention’s cost structure.

A further problem of calculating programme costs is the exchange rate to foreign currencies. Many of the francophone West African countries are part of monetary unions (BEAC, BCEAO) that use currencies which are pegged to the Euro. Exchange rate fluctuations between Euro and US Dollar may have significant impact on project costs, making non-European Union financed projects more or less attractive for donor organizations. Project costs may hence vary depending on where the project is run and from which sources *Bti* and other material are obtained. A further limitation to this study is the application of a 50 % treatment threshold for larviciding. In the present study, this threshold was deliberately set to gather data on the achievable reduction with only the most productive half of water bodies treated. From an epidemiological point of view, it would be rather advisable to not work with an a priori set threshold of treated water bodies but an absolute threshold of larval densities, which if exceeded, indicates the need for treatment. For many settings it could turn out that larval productivity is still high although the breeding sites belong to the half with the lower larval densities.

Several assumptions based on field experience were introduced to calculate needed programme resources. The number of required spray personnel was set a priori to one person per village based on the observations of a low number of vector breeding sites in most of the villages. Despite the technically sufficient workforce it may be generally an advantage to employ sprayers as teams of two or more to increase motivation and backup security in the case of illness or absence. An increase in personnel would not significantly increase cost for wages on an hourly basis but it would double the need for spraying equipment.

The authors are aware that the introduction of our *Bti* cost-model comes with a considerable degree of generalization. While this feature makes the model easily applicable for other settings, at the same time, it comes at cost of context-dependent prediction accuracy. Both goals are not achievable without accepting certain trade-offs. Thus, it is worth mentioning that our cost-model outcomes only give a proxy for per capita programme costs and are expected to deliver more realistic results for settings that resemble the study region it was developed in. Nonetheless, the authors are confident that this cost-model not only sheds light into the cost structure of LSM programmes but that it will serve as a helpful implementation tool for larviciding programmes elsewhere by providing reliable cost estimates on technical, infrastructural and personnel needs.

## Conclusions

Larvicide-based LSM is an additional, complementary tool for malaria control programmes that so far did not receive the attention it deserves for designing national and international policies. Particularly, in combination with LLINs and indoor residual spraying it proved to be a highly effective malaria vector control measure. For selected environments the use of remote sensing derived risk maps might be a promising approach to reduce the number of treated water bodies while, at the same time, keeping programme costs at reasonably low levels. Although today's WHO recommendations promote the use of LSM mainly for urban areas with high population densities with the underlying idea of obtaining reduced costs per person, rural areas should not be considered a priori as ineligible for spraying interventions. Given the continuously adapting nature of malaria vector mosquitoes to insecticides, we make a case to shift more attention to hereof unaffected control strategies such as *Bti* based LSM. The latter might work as an important complementary tool to achieve the ambitious goals pursued by the WHO's global technical strategy for malaria between 2016 and 2030, to reduce malaria incidence and mortality by at least 90 % [1].

## Additional file

10.1186/s12936-016-1438-8 Total accrued costs in US$ for the EMIRA research project. Calculations based on 2013 costs for material and personnel using a 2013–2015 average exchange rate of US$ 1 = € 0.82.
