# Supplementary material for: Routine implementation costs of larviciding with Bacillus thuringiensis israelensis against malaria vectors in a district in rural Burkina Faso
Source: Malar J. 2016 Jul 22;15:380. doi: 10.1186/s12936-016-1438-8 (PMC4957841; doi:10.1186/s12936-016-1438-8)
Supplement: Supplementary file 1 — 10.1186/s12936-016-1438-8 Total accrued costs in US$ for the EMIRA research project. Calculations based on 2013 costs for material and personnel using a 2013–2015 average exchange rate of US$ 1 = € 0.82. [file 12936_2016_1438_MOESM1_ESM.docx]

Additional file 1: Table S1. Total accrued costs in US$ for the EMIRA research project. Calculations based on 2013 costs for material and personnel using a 2013 to 2015 average exchange rate of US$1 = € 0.82

|  | **Baseline year (no intervention)** | | | | **Intervention year 1** | | | | **Intervention year 2** | | | |  |
| --- | --- | --- | --- | --- | --- | --- | --- | --- | --- | --- | --- | --- | --- |
|  | ***Persons*** | ***Cost/month*** | ***Months*** | ***Total*** | ***Persons*** | ***Cost/month*** | ***Months*** | ***Total*** | ***Persons*** | ***Cost/month*** | ***Months*** | ***Total*** | **TOTAL** |
| **Personnel** |  |  |  |  |  |  |  |  |  |  |  |  |  |
| Project manager Germany ^1^ | 1 | 4,570 | 12 | 54,840 | 1 | 4,570 | 12 | 54,840 | 1 | 4,570 | 12 | 54,840 | 164,520 |
| Project manager Burkina Faso | 1 | 1,220 | 12 | 14,640 | 1 | 1,220 | 12 | 14,640 | 1 | 1,220 | 12 | 14,640 | 43,920 |
| LSM specialist consultant | 1 | 7,300 | 1 | 7,300 | 1 | 7,300 | 1 | 7,300 | 1 | 7,300 | 1 | 7,300 | 21,900 |
| Entomologic technicians | 2 | 390 | 12 | 9,360 | 2 | 390 | 12 | 9,360 | 2 | 390 | 12 | 9,360 | 28,080 |
| GIS specialist ² |  |  |  |  |  |  |  |  |  |  |  |  | 0 |
| Larviciding personnel |  |  |  |  | 180 | 30 | 5 | 27,000 | 180 | 30 | 5 | 27,000 | 54,000 |
| Larviciding supervisors |  |  |  |  | 15 | 62 | 5 | 4,650 | 15 | 62 | 5 | 4,650 | 9,300 |
| **SUBTOTAL** |  |  |  | 86,140 |  |  |  | 117,790 |  |  |  | 117,790 | **321,720** |
| **Consumables** | ***Amount*** | ***Unit cost*** |  | ***Total*** | ***Amount*** | ***Unit cost*** |  | ***Total*** | ***Amount*** | ***Unit cost*** |  | ***Total*** |  |
| Larvicide |  |  |  |  | 1200kg | 40 |  | 48,000 | 600kg | 42 |  | 25,080 | 73,080 |
| **SUBTOTAL** |  |  |  |  |  |  |  | 48,000 |  |  |  | 25,080 | **73,080** |
| **Transport and freight costs** |  |  |  |  |  |  |  |  |  |  |  |  |  |
| Transport costs fieldwork | 1 | 2,200 |  | 2,200 | 1 | 3,800 |  | 3,800 | 1 | 3,950 |  | 3,950 | 9,950 |
| Air freight larvicide |  |  |  |  | 1 | 12,000 |  | 12,000 | 1 | 9,000 |  | 9,000 | 21,000 |
| Air freight knapsack sprayers |  |  |  |  | 1 | 7,000 |  | 7,000 |  |  |  |  | 7,000 |
| Air freight light traps | 1 | 2,100 |  | 2,100 |  |  |  |  |  |  |  |  | 2,100 |
| Travel and accomodation costs | 2 | 1,200 |  | 2,400 | 2 | 1,200 |  | 2,400 | 2 | 1,200 |  | 2,400 | 7,200 |
| **SUBTOTAL** |  |  |  | 6,700 |  |  |  | 25,200 |  |  |  | 15,350 | **47,250** |
| **Activities** | ***Rounds*** | ***Cost/round*** |  | ***Total*** | ***Rounds*** | ***Cost/round*** |  | ***Total*** | ***Rounds*** | ***Cost/round*** |  | ***Total*** |  |
| Staff training events | 2 | 420 |  | 840 | 2 | 1,600 |  | 3,200 | 2 | 840 |  | 1,680 | 5,720 |
| Community sensitization |  |  |  |  | 1 | 1,950 |  | 1,950 |  |  |  | 0 | 1,950 |
| Perception study on larviciding | 1 |  |  |  |  |  |  |  |  |  |  | 2,440 | 2,440 |
| Testing for parasitemia per round | 2 | 1,220 |  | 2,440 | 2 | 1,220 |  | 2,440 | 2 | 1,220 |  | 2,440 | 7,320 |
| Mapping of larval sources | 1 | 1,040 |  | 1,040 |  |  |  | 0 |  |  |  | 0 | 1,040 |
| Mapping for risk map creation | 1 | 2,200 |  | 2,200 |  |  |  | 0 |  |  |  | 0 | 2,200 |
| Mortality & Morbidity data collection | 1 | 1,830 |  | 1,830 | 1 | 1,830 |  | 1,830 | 1 | 1,830 |  | 1,830 | 5,490 |
| **SUBTOTAL** |  |  |  | 8,350 |  |  |  | 9,420 |  |  |  | 8,390 | **26,160** |
| **Capital costs** | ***Amount*** | ***Unit cost*** |  | ***Total*** | ***Amount*** | ***Unit cost*** |  | ***Total*** | ***Amount*** | ***Unit cost*** |  | ***Total*** |  |
| Knapsack sprayers |  |  |  |  | 180 | 173 |  | 31,140 |  |  |  |  | 31,140 |
| CDC lighttraps, batteries | 40 | 150 |  | 6,000 |  |  |  |  |  |  |  |  | 6,000 |
| GPS, Computer | 6 | 330 |  | 2,000 |  |  |  |  |  |  |  |  | 2,000 |
| Satellite imagery | 2 | 610 |  | 1,220 |  |  |  |  |  |  |  |  | 1,220 |
| Laboratory equipment | 1 | 275 |  | 275 |  |  |  |  |  |  |  |  | 275 |
| Protective clothes |  |  |  |  | 180 | 20 |  | 3,600 |  |  |  |  | 3,600 |
| **SUBTOTAL** |  |  |  | 9,495 |  |  |  | 34,740 |  |  |  |  | **44,235** |
| **TOTAL** |  |  |  | 110,685 |  |  |  | 235,150 |  |  |  | 166,610 | **512,445** |
| Overheads 10% |  |  |  | 11,069 |  |  |  | 23,515 |  |  |  | 16,661 | 51,245 |
| **TOTAL PROGRAM COSTS** |  |  |  | **121,754** |  |  |  | **258,665** |  |  |  | **183,271** | **563,690** |

1: Part time salary following German university pay schemes.
2: Salary for a GIS specialist is not listed here since this task was covered by the project management in Germany
